# Inclusive Leadership in Disadvantaged Neighbourhoods: A Qualitative Analysis of Stakeholder Perspectives on Its Potential Role in Academic Achievement

**DOI:** 10.3390/bs16081336

**Published:** 2026-08-03

**Authors:** Gülenaz Selçuk, Ayşegül Ayyıldız Asil

**Affiliations:** 1 Faculty of Sports Sciences, Manisa Celal Bayar University, Manisa 45040, Turkey; gulenaz.selcuk@cbu.edu.tr; 2 Faculty of Education, Hakkari University, Hakkari 30000, Turkey

**Keywords:** inclusive leadership, disadvantaged, achievement, qualitative, perspective

## Abstract

The aim of this study is to examine the potential role of inclusive leadership in disadvantaged neighbourhoods. A qualitative research method and case study were employed in the study. The study group consists of 53 participants. A semi-structured interview form was used, and the findings were analyzed through content analysis. In the study, 5 themes, 20 codes, and 34 sub-codes were created. According to the findings, first, inclusive leadership was predominantly conceptualised through the principles of equality, fairness, and participation, with stakeholders emphasising equal treatment, inclusion of all individuals in school processes, and participatory decision-making. While school administrators tended to view inclusion from a structural and managerial perspective, teachers emphasised its relational, emotional, and communicative dimensions. Second, the cultural structure of disadvantaged neighbourhoods was characterised primarily by low socioeconomic conditions, strong traditional values, and cultural diversity, creating both challenges and opportunities for inclusive leadership practices. Third, school administrators’ inclusive leadership practices were largely perceived positively and reflected instructional leadership, open communication, stakeholder participation, attention to individual differences, collaboration, and fairness, although weaknesses related to empathy and engagement with external stakeholders were also identified. Fourth, inclusive leadership was perceived to contribute to academic achievement indirectly through enhanced student motivation, school belonging, organisational trust, positive school climate, cooperation, and respect for individual differences. Finally, participants identified family disengagement, economic hardship, inadequate physical resources, and limited community support as major barriers to inclusive leadership, while recommending stronger family-school collaboration, increased community involvement, leadership training, and structural improvements as key strategies for enhancing both inclusion and academic success. Overall, the findings suggest that the influence of inclusive leadership on academic achievement in disadvantaged contexts operates through both structural and relational mechanisms and is strongly shaped by broader socioeconomic and community conditions.

## 1. Introduction

Inclusive leadership originated within organisational and management research and has been associated with leaders’ openness, accessibility, and availability toward followers, as well as the creation of psychologically safe environments that encourage participation, learning, and collaboration (Nembhard & Edmondson, 2006; Carmeli et al., 2010). More recent conceptualizations emphasize employees’ sense of belongingness while simultaneously valuing their unique contributions, highlighting inclusion as a process through which individuals feel both accepted and appreciated within organizations (Shore et al., 2011; Randel et al., 2018). Inclusive leadership has increasingly been recognized as a mechanism for promoting engagement, trust, and collective effectiveness across diverse organisational settings. In educational settings, inclusive leadership extends beyond organisational participation and encompasses equitable access to learning opportunities, responsiveness to cultural diversity, and advocacy for communities (Ryan, 2006b; Khalifa et al., 2016). School leaders are expected not only to manage educational institutions but also to challenge structural barriers that limit educational opportunities and student success. Inclusive leadership has become closely associated with broader approaches such as culturally responsive leadership, social justice leadership, and equity-oriented leadership. Despite increased access to schooling, persistent achievement gaps highlight the need for leadership approaches that address the unique disadvantages faced by students in disadvantaged contexts and ensure equitable learning outcomes (Tan & Gümüş, 2024). Traditional school improvement models have often proven insufficient in mitigating these disparities, underscoring the urgent need for innovative practices such as culturally sustaining leadership (Santamaría & Santamaría, 2016). This leadership paradigm, encompassing culturally responsive and equity-oriented approaches, is crucial for disrupting normative structures that perpetuate educational inequities and for affirming the cultural assets and lived experiences of historically underrepresented communities (White et al., 2025). This comprehensive approach to leadership aims to transform the entire school environment, making it responsive to the diverse needs of minoritized students by centering inclusion, advocacy, and social justice (Khalifa et al., 2016). This involves recognizing that social justice leadership constitutes an ontological stance, which manifests in varied interpretations and practices across different educational contexts (Chaaban et al., 2025). Different perspectives and limited evidence in the literature highlight the need for empirical research examining the role of inclusive leadership practices in academic achievement, particularly in disadvantaged school contexts (Karaköse et al., 2023; Leithwood, 2021).

The literature indicates that certain leadership styles indirectly affect student learning and academic performance in contexts characterized by socioeconomic challenges; accordingly (Maqbool et al., 2023; Ucar & Dalgic, 2021), the current study addresses a critical gap in the existing literature. Furthermore, research goes beyond generalised notions of leadership by identifying specific practices that promote equity in education in disadvantaged communities, thereby revealing the nuanced effects of leadership on student achievement (Tan et al., 2021). Studies quantitatively analyze student achievement outcomes in such educational settings, highlighting leadership behaviours that emphasize cultural responsiveness, community engagement, and systemic equity (Mansfield & Lambrinou, 2024; Washington & Johnson, 2023). This is particularly relevant given the increasing resistance to diversity, equity, and inclusion initiatives in educational settings, making it imperative to understand how leadership adapts to and navigates such challenges to promote student success (Blackledge, 2025). The persistent achievement gap between various racial and socioeconomic groups necessitates a comprehensive understanding of leadership practices that move beyond superficial interventions to address systemic inequities (Nadrozny, 2017).

Although previous studies have demonstrated associations between leadership practices and student outcomes, less attention has been paid to how inclusive leadership is understood and enacted by stakeholders within disadvantaged school communities. Existing research has predominantly focused on school leaders or teachers separately, providing limited insight into how different stakeholder groups collectively interpret inclusive leadership and its perceived potential role in academic achievement. The theoretical foundation of the study is based on the inclusive leadership approach developed by Nembhard and Edmondson (2006). This approach suggests that leaders’ inclusive behaviours, which encourage employees to express their ideas, enhance perceptions of psychological safety within organizations, thereby strengthening learning, collaboration, and improvement-oriented behaviours. When adapted to the educational context, this indicates that school principals’ inclusive leadership behaviours may influence teachers’ pedagogical practices and student learning processes.

In this regard, the study aims to examine inclusive leadership within the interaction of school culture, leadership practices, and the socio-cultural structure of disadvantaged neighbourhoods. Through the perspectives of school principals, teachers, and local community representatives, the study will analyze inclusive leadership in a multidimensional manner, focusing on definitions of inclusive leadership, the cultural structure of the school’s surrounding environment, inclusive leadership behaviours, its potential role in academic achievement, barriers to inclusive leadership, and recommendations for its improvement. In this context, the study seeks to reveal how the inclusive leadership approach is associated with academic achievement in disadvantaged neighbourhoods.

### 1.1. Literature Background

#### 1.1.1. Inclusive Leadership: Conceptual Development

The concept of inclusive leadership gained prominence in the organisational and management literature through the work of Nembhard and Edmondson (2006), who defined leader inclusiveness as behaviours through which leaders invite and appreciate the contributions of others. Although the inclusive leadership approach was originally developed in the context of teams working in the healthcare sector (Nembhard & Edmondson, 2006), the core elements of the model—such as psychological safety, participation, collaboration, and encouraging the expression of diverse viewpoints—are also considered important in educational organizations. Their study highlighted the role of inclusive leader behaviours in fostering psychological safety and encouraging learning, collaboration, and improvement-oriented behaviours. As organizations became increasingly diverse, inclusive leadership emerged as an important approach for creating environments where individuals are encouraged to participate, share their perspectives, and contribute to collective goals.

Subsequent research expanded the conceptualization of inclusive leadership. Carmeli et al. (2010) emphasised the importance of leaders’ interactions with followers and identified specific behavioral characteristics associated with inclusive leadership. More recent studies further broadened the concept by linking it to employees’ experiences of inclusion, belongingness, and recognition within organizations (Shore et al., 2011; Randel et al., 2018). Consequently, inclusive leadership has evolved from a focus on leader behaviours to a broader framework emphasizing participation, engagement, and inclusion within diverse organisational settings. Beyond leader behaviours, inclusive leadership has also been conceptualized as a relational and values-based approach that emphasizes respect, participation, and the active involvement of organisational members in collective processes. According to Booysen (2014), inclusive leadership extends beyond managing diversity and seeks to create organisational cultures in which individuals feel genuinely included, respected, and empowered to contribute.

#### 1.1.2. Dimensions of Inclusive Leadership

The literature identifies several core dimensions of inclusive leadership. According to Carmeli et al. (2010), inclusive leadership is characterized by openness, accessibility, and availability. Openness refers to leaders’ willingness to consider different viewpoints and encourage the expression of ideas. Accessibility reflects leaders’ readiness to engage in communication and interaction with organisational members, while availability denotes their responsiveness to followers’ concerns and needs.

In addition to these behavioral dimensions, contemporary perspectives emphasize the importance of inclusion as a social experience. Shore et al. (2011) conceptualized inclusion as the simultaneous experience of belongingness and uniqueness. Building on this perspective, Randel et al. (2018) argued that inclusive leadership promotes positive outcomes when individuals feel accepted as valued members of an organization while also having their unique contributions recognized and appreciated. Together, these dimensions contribute to psychologically safe environments that support collaboration, learning, innovation, and organisational effectiveness.

#### 1.1.3. Inclusive Leadership and Related Leadership Models

Inclusive leadership shares several common principles with other leadership approaches frequently discussed in educational research. One such approach is culturally responsive leadership, which focuses on recognizing, valuing, and integrating students’ cultural identities and experiences into educational practices (Khalifa et al., 2016). Another related framework is social justice leadership, which emphasizes addressing structural inequalities and advocating for disadvantaged individuals and groups within educational systems (Ryan, 2006b).

Although these approaches differ in emphasis, they all seek to promote equity and improve opportunities for underserved populations. Inclusive leadership is distinguished by its particular focus on participation, collaboration, stakeholder voice, and the creation of psychologically safe environments in which diverse perspectives are welcomed and valued. Therefore, inclusive leadership can be viewed as a complementary framework that intersects with culturally responsive and social justice leadership while maintaining its own distinct focus on inclusion and participation.

#### 1.1.4. Inclusive Leadership and Academic Achievement

Inclusive school environments are associated with stronger stakeholder engagement and a greater sense of belonging among students. According to Echols (2009), schools that foster inclusive cultures encourage participation and collaboration among members of the school community, creating conditions that may support student engagement and learning. In schools, inclusive leadership aims to safeguard the interests of all stakeholders and creates suitable conditions for effective communication processes, where respect for values, beliefs, and lifestyles is considered an integral part of education (Dorczak, 2011; Ryan, 2006b). This approach plays an important role in enabling school leaders to create more inclusive environments for both teachers and students (Garrison-Wade et al., 2007). It also involves paying attention to individuals who are at risk of exclusion, encouraging communication with them, and supporting their participation in decision-making processes (Ryan, 2006a).

It is vital for developing an educational environment in which culturally responsive leadership can effectively bridge the cultural gap between diverse student groups and educators, thereby reducing feelings of disengagement and promoting academic achievement (Olanrewaju & Omeghie, 2024). Jayavant’s (2016) study focuses on quantitatively measuring the impact of dimensions of inclusive leadership such as collaborative governance and culturally competent practices on the academic performance of students in disadvantaged urban schools. In the study by Bao (2024), a deeper understanding was developed of how school principals’ inclusive leadership influences teachers’ innovative behaviours and the overall school innovation climate, which is critical for addressing the challenges of rapidly evolving educational environments. Vem et al. (2024) indicate that psychological empowerment enhances innovative teaching practices among academics; in this process, achievement orientation plays a mediating role, while inclusive leadership plays a moderating role.

### 1.2. Research Gap and the Current Study

Existing studies demonstrate the importance of inclusive leadership in the educational context from various perspectives. For example, Jayavant (2016) focused on quantitatively examining the impact of dimensions of inclusive leadership, such as collaborative governance and cultural competence, on student achievement in disadvantaged urban schools. Bao (2024) investigated the effects of school principals’ inclusive leadership on teachers’ innovative behaviours and the overall school innovation climate. Vem et al. (2024) addressed the relationships between psychological empowerment, achievement orientation, and inclusive leadership in the context of innovative academic teaching. In addition, culturally responsive leadership is emphasised as a factor that can reduce cultural gaps between students and educators, thereby supporting academic achievement.

Taken together, prior research has established that inclusive leadership is associated with a range of positive educational outcomes, including academic achievement, innovation, teacher development, and school climate. However, three important limitations remain. First, much of the existing evidence is derived from quantitative studies that identify relationships between variables but provide limited insight into how inclusive leadership shapes educational outcomes in practice. Second, previous studies have predominantly focused on the perceptions of a single stakeholder group, such as principals or teachers, while overlooking the perspectives of other actors involved in local educational processes. Third, relatively little is known about how inclusive leadership operates within disadvantaged school communities, where socioeconomic challenges, cultural diversity, and limited educational resources may shape both leadership practices and achievement outcomes. Accordingly, the present study does not seek to establish whether inclusive leadership is associated with academic achievement, as this relationship has already been highlighted in previous research. Rather, it seeks to explore the perceived mechanisms through which inclusive leadership may contribute to educational outcomes in disadvantaged school contexts and how these mechanisms are interpreted by different stakeholder groups, including school administrators, teachers, and local community representatives. Although inclusive leadership shares several common principles with culturally responsive leadership and social justice leadership, important conceptual distinctions remain. Culturally responsive leadership primarily focuses on recognizing, valuing, and integrating students’ cultural backgrounds into educational practices (Khalifa et al., 2016), whereas social justice leadership emphasizes challenging systemic inequities and promoting equity-oriented change (Ryan, 2006b). Inclusive leadership, in contrast, places greater emphasis on participation, belonging, accessibility, and the active involvement of diverse stakeholders in decision-making processes (Nembhard & Edmondson, 2006). Despite these conceptual overlaps, limited research has examined how these dimensions are interpreted and experienced by different stakeholder groups within disadvantaged school communities.

The aim of this study is to examine inclusive leadership in disadvantaged neighbourhoods.

Research Questions

RQ1. How do teachers, school administrators, and local community representatives define and conceptualise inclusive leadership in disadvantaged school contexts?
RQ2. What are the characteristics of the cultural structure of disadvantaged neighbourhoods?
RQ3. What inclusive leadership behaviours are exhibited by school leaders in disadvantaged school settings?
RQ4. How is inclusive leadership perceived to shape students’ academic achievement in disadvantaged neighbourhoods?
RQ5. What barriers hinder the implementation of inclusive leadership practices, and what strategies are suggested to strengthen inclusive leadership in disadvantaged school contexts?

## 2. Materials and Methods

A qualitative research method was chosen. Qualitative research, which uses qualitative data collection methods such as observation, interviews, and document analysis to solve a problem, represents a subjective-interpretive process aimed at perceiving previously known or unnoticed problems and realistically addressing natural phenomena related to the problem (Seale, 1999). A qualitative research method was chosen because the study aimed to explore in depth how inclusive leadership shapes academic achievement in disadvantaged school contexts and how these experiences are perceived by different stakeholders.

### 2.1. Design

This study employed a case study design, a qualitative research method. A case study systematically investigates a limited number of situations in a given field in order to create the infrastructure for their development (Chmiliar, 2010). Generally, a case study is a method used when the researcher has little influence over the events and when examining the questions of why and how (Yin, 2003). A case study design was employed because it enabled an in-depth examination of inclusive leadership practices and their perceived potential role in academic achievement within the specific context of disadvantaged school communities.

### 2.2. Study Group

The participants in the study were selected using maximum diversity and snowball sampling techniques, which are among the purposeful sampling methods. Maximum variation sampling was used to ensure the inclusion of participants with diverse professional roles and experiences related to the research topic. Diversity criteria included stakeholder group (school administrator, teacher, and local community representative), school level (primary, secondary, and high school), gender, professional seniority, and length of service in the neighbourhood. After identifying participants who met these diversity criteria, snowball sampling was used to reach additional participants who had relevant experience and could provide rich information regarding inclusive leadership practices in disadvantaged school settings. Thus, maximum variation sampling guided the initial selection of participants, while snowball sampling facilitated access to further information-rich cases. Accordingly, teachers, administrators and local community representatives associated with 22 schools (5 primary schools, 7 secondary schools, and 10 high schools) serving in Yunus Emre District of Manisa were included in the research. The study group consists of 53 participants. The neighbourhood was selected based on indicators commonly associated with educational disadvantage, including low socioeconomic status, high levels of unemployment, limited educational resources, low parental educational attainment, and school records indicating relatively low academic achievement compared with district averages. In addition, local educational authorities and school administrators identified the area as socioeconomically disadvantaged. Since the study was conducted within a qualitative research paradigm, the aim was not statistical generalization but obtaining an in-depth understanding of inclusive leadership practices within a specific disadvantaged educational context. Therefore, the findings should be interpreted as context-bound and analytically transferable rather than statistically generalizable. Although the local community representative group consisted of only five participants, these individuals were selected because of their direct engagement with schools and their active role in the local educational ecosystem. The inclusion of teachers, school administrators, and local community representatives was intended to capture stakeholder perspectives; however, the findings should not be interpreted as representing all stakeholder groups within disadvantaged communities. Maximum diversity sampling aims to provide an in-depth understanding of the phenomenon from different perspectives by including individuals with characteristics that may reflect different viewpoints on the subject (Patton, 2014). In the snowball sampling method, the researcher begins by identifying the first few individuals who have knowledge of the subject and can participate in the research. These initial participants then refer other potential participants with similar characteristics or experiences in their environment to the researcher. This chain of referrals continues until the sample is expanded and the research objectives are achieved (Atkinson & Flint, 2001). The study group consisted exclusively of adult stakeholders who were directly involved in the educational processes of schools located in disadvantaged neighbourhoods. Participants included school administrators, teachers, and local community representatives. These groups were selected because of their professional and social engagement with the schools and their potential to provide in-depth insights into leadership practices and their perceived influence on students’ educational experiences. As the study did not include students or parents, the findings should be interpreted as reflecting adult stakeholders’ perceptions of inclusive leadership and its perceived potential role to academic achievement rather than direct measures of student outcomes. The chosen research context was not selected with the aim of representing all disadvantaged communities in Türkiye. Instead, it was chosen as a knowledge-rich situation where socioeconomic disadvantages are prevalent, educational resources are limited, and school-community interactions are prominent. Therefore, this context is of particular importance in terms of its potential to reveal how inclusive leadership practices are perceived and experienced under conditions of structural inequalities and disadvantage. The demographic characteristics of the participants are listed below:

According to Table 1, participants’ gender distribution shows that 16 are female and 10 are male. When the duration of service in the neighbourhood is examined, the highest frequency is in the 1–5 year range (10 participants), followed by the 6–10 year range (9 participants). The number of participants who have served for 11–15 years is quite low (2 participants), while 5 participants have been working in the same neighbourhood for 16 years or more. In terms of professional seniority, the most striking finding is the high number of teachers with 16 years or more of experience (17 participants). There are 6 participants with 11–15 years of experience, while only 2 participants fall within the 6–10 year range and just 1 participant within the 1–5 year range. Regarding subject area distribution, the highest participation comes from Turkish language teachers (6 participants). Science, Guidance and Psychological Counseling, and Technology each have 4 participants. Mathematics is represented by 3 participants, while Social Studies has a more limited representation with 2 participants. Primary classroom teaching, Physical Education and Sports, and English teaching each have only 1 participant, making them the least represented fields.

According to Table 2, the distribution of participants according to the period they have served in the neighbourhood shows that the majority have relatively shorter service durations. All participants are male. Specifically, 2 participants have served for 1–5 years, while the remaining categories of 6–10 years, 11–15 years, and 16 years and above are each represented by 1 participant. In terms of educational status, most participants hold an undergraduate degree (3 participants), while 2 participants have completed postgraduate education. Regarding job roles, the data reveal that the majority of participants are religious officials (4 participants), with only 1 participant serving as an NGO president.

According to Table 3; the gender distribution of the participants indicates that 13 are male and 9 are female. When the period served in the neighbourhood is examined, the majority of participants have worked there for 1–5 years (14 participants). This is followed by 6–10 years (6 participants) and 11–15 years (2 participants). In terms of seniority, the distribution appears more balanced across categories. There are 6 participants with 1–5 years of experience, 5 with 6–10 years, 7 with 11–15 years, and 4 with 16 years and above. Regarding school type, the largest group of participants works in high schools (10 participants), followed by secondary schools (7 participants) and primary schools (5 participants).

### 2.3. Data Collection

In qualitative studies, interviews can be classified as unstructured, semi-structured, and structured according to their structural characteristics such as the standardization of question preparation, organization, sequencing, and posed questions; they can also be named differently according to whether they focus on a group or individuals, such as a focus group interview or an in-depth interview, or according to the technical method of conducting the interview, such as face-to-face, telephone, or online interview (Creswell, 2018; Kvale & Brinkmann, 2009; Merriam & Tisdell, 2015). The most commonly used technique in qualitative research is semi-structured interviews (Patten & Newhart, 2018). A key feature of a semi-structured interview is keeping the number of basic interview questions quite limited and deriving follow-up and final questions from them (Roulston, 2010; Rubin & Rubin, 2012).

Within the scope of the study, data were collected through a semi-structured interview form on inclusive leadership behaviours in disadvantaged schools. In the development process of the interview form, a comprehensive literature review on inclusive leadership was first conducted. Based on this review, five main themes were identified: the definition of inclusive leadership, the cultural structure of the school environment, inclusive leadership behaviours, the potential role of inclusive leadership in academic achievement, and the problems and solutions related to inclusive leadership practices. These themes were selected in alignment with both the dimensions frequently addressed in the literature and the aims and research questions of the study.

Following the identification of the themes, open-ended questions were developed for each theme to elicit participants’ experiences in depth. The initial draft of the interview form was reviewed by two experts in educational sciences and qualitative research. The experts evaluated the form in terms of content validity, clarity, and alignment with the research questions.

In order to test the applicability of the interview form, a pilot study was conducted with one teacher, one school administrator, and one local community representative. Following the pilot application, minor revisions were made where necessary. Participants in the pilot study were not included in the main sample or in the data analysis.

The interview form consists of two sections. The first section includes demographic information about the participants, while the second section contains open-ended questions related to the research themes. The interview form was specifically developed for this study and has not been used in any previous research. Data were collected through face-to-face interviews. Interviews lasted between 35 and 60 min, with an average duration of approximately 45 min. All interviews were conducted in locations preferred by the participants to ensure comfort and confidentiality. Data collection and analysis were conducted concurrently. During the later stages of data collection, no substantially new codes or themes emerged from the interviews. This recurrence of themes across participant groups was considered an indication of thematic saturation, and data collection was concluded at this point. Given the potentially sensitive nature of leadership-related issues, participants were assured that there were no right or wrong answers and that all responses would be anonymized. This approach was intended to reduce social desirability bias and encourage participants to express their views openly. Given the potentially sensitive nature of some topics, particularly those involving criticism of school leadership and administrative practices, participants were informed that their responses would remain confidential and would not be shared with school authorities or other participants. Interviews were conducted individually in a setting chosen by the participants, and all identifying information was removed during transcription to encourage open and honest responses.

### 2.4. Data Analysis

Content analysis was preferred in the research. Content analysis was used to systematically identify and interpret recurring themes, patterns, and stakeholder perspectives regarding inclusive leadership and its potential role in academic achievement. Content analysis is one of the important methods of research techniques in the social sciences. This technique is defined as a systematic, methodological and objective method used to identify, classify and interpret the basic components in the content of a text/discourse; it is carried out to analyze the presence of systematically determined categories/codes within a text or visual (Robert & Bouillaguet, 1997).

Content analysis was conducted following a step-by-step procedure:

All interview recordings were transcribed verbatim.

Data were anonymized and participants were coded as for teachers: T1–T26, administrators: A1–A22, and local community representatives: L1–L5.

Identifiable information was removed to ensure confidentiality.

Meaningful expressions were identified and subjected to open coding.

Similar codes were grouped under subcategories based on conceptual similarity.

Subcategories were further organized into broader categories and themes in line with the research questions.

The coding process was carried out with the MAXQDA 2020 software package. The coding process was iterative. Initial open coding was conducted independently by the two authors. The resulting codes were compared and discussed until consensus was reached. To enhance credibility, an independent researcher with expertise in qualitative research reviewed a subset of the transcripts, the coding framework, and thematic interpretations. Thus, three individuals were involved in the analytic process: the two authors as primary coders and one independent researcher as an external reviewer. To minimize researcher bias, the researchers maintained reflective notes throughout the research process and continuously compared emerging interpretations with the original interview data. In addition, coding decisions and thematic interpretations were reviewed by an independent researcher. A subset of transcripts was independently coded by an independent researcher. Coding discrepancies were discussed until consensus was reached and the coding framework was refined accordingly. An audit trail was maintained throughout the analysis process, including records of coding decisions, code revisions, theme development, analytic memos, and discussions between researchers. This documentation enabled the tracking of how themes evolved from the raw data and enhanced the transparency and confirmability of the findings.

Throughout the study, the researchers engaged in reflexive practices by maintaining reflective journals and discussing how their professional backgrounds, assumptions, and prior experiences with educational leadership might influence data collection and interpretation. These reflections were revisited during the coding and analysis process in an effort to minimize potential bias.

Guba and Lincoln (1982) proposed the concept of “credibility” instead of validity and reliability in qualitative research, and grouped it under four main headings: trustworthiness, reliability, confirmability, and transferability. For credibility, long-term interaction was conducted, and participant confirmation and expert opinions were obtained. To enhance the trustworthiness of the analysis, an independent researcher reviewed the coding process. A subset of the interview transcripts was coded independently by both researchers, and the resulting codes were compared. Any discrepancies were discussed until consensus was reached, and the coding framework was refined accordingly. This process contributed to the consistency and credibility of the thematic analysis. In confirmability, findings based on participant statements were presented in the research through direct quotations. Transferability allowed for the adaptability of findings to similar situations. To increase the reliability of the research, the purpose and method of the research were explained to the participants in detail. Throughout the study, the researchers engaged in reflexive practices by maintaining reflective journals and critically examining how their professional backgrounds, assumptions, and prior experiences with educational leadership might shape data collection and interpretation. Particular attention was given to potential power dynamics between researchers and participants, especially given the study’s focus on disadvantaged communities. The researchers sought to minimize these influences by encouraging open dialogue, emphasizing voluntary participation, and continuously reflecting on how their own perspectives might affect the interpretation of participants’ accounts.

To enhance the credibility of the analysis, a subset of the interview transcripts was independently coded by an external researcher. Inter-coder agreement was calculated using percentage agreement. The comparison of coding decisions yielded an agreement rate of 80%, indicating a high level of consistency between coders. Discrepancies were discussed until consensus was reached and the coding framework was refined accordingly.

## 3. Results

According to Table 4, teachers most frequently described inclusive leadership as “a leader who treats everyone equally” (f = 6), “a leader who involves everyone in decision-making” (f = 4), and “a leader who meets all needs” (f = 4). These findings suggest that teachers primarily conceptualize inclusive leadership in terms of equality, fairness, and participation. Regarding inclusive leadership practices, the strongest emphasis was placed on the instructional leadership dimension (f = 10), followed by facilitating communication (f = 5) and taking individual differences into account (f = 4). According to teachers, the most significant contribution of inclusive leadership to academic achievement is fostering happy and motivated students (f = 12). They also believe that inclusive leadership enhances organizational trust and promotes more positive feelings toward school. On the other hand, teachers identified parental lack of interest (f = 12), students’ lack of engagement and inattentiveness (f = 5), and communication problems with parents (f = 4) as the main barriers to the effective implementation of inclusive leadership. Among the proposed solutions, providing inclusive leadership training for school administrators (f = 8) emerged as the most frequently mentioned recommendation. Overall, teachers perceive inclusive leadership as an important leadership approach that strengthens student motivation and contributes positively to the school climate.

According to Table 5, school administrators most frequently described inclusive leadership as “a leader who includes everyone in the system” (f = 9) and “a leader who treats everyone equally” (f = 7). They characterized the neighborhood structure primarily as having a low socioeconomic status (f = 9) and being tradition-oriented (f = 6). Among inclusive leadership practices, participation in decision-making processes (f = 6), fairness (f = 5), and facilitating communication (f = 5) emerged as the most prominent practices. Regarding the potential outcomes of inclusive leadership, school administrators most frequently emphasized increased organizational trust (f = 9), the development of positive feelings toward school (f = 7), and the presence of happy and motivated students (f = 7). Among the proposed solutions, increasing support from local authorities (f = 13) and organizing family-based training programs (f = 7) were highlighted most frequently. In contrast, parental lack of interest (f = 7) and communication problems with parents (f = 4) were identified as the main challenges. These findings suggest that school administrators largely associate the success of inclusive leadership with the support of stakeholders outside the school, particularly families and local authorities.

According to Table 6, local community representatives most frequently described inclusive leadership as “a leader who treats everyone equally” (f = 2), while also emphasizing the importance of involving everyone in decision-making processes (f = 1) and including all stakeholders within the system (f = 1). These findings indicate that local authorities primarily associate inclusive leadership with equality, participation, and broad stakeholder inclusion. Regarding the neighborhood context, they characterized the area as having a low socioeconomic status (f = 2) and a tradition-oriented structure (f = 1), highlighting the socioeconomic and cultural challenges that may affect educational processes. Among the challenges related to inclusive leadership practices, local authorities most frequently pointed to weak public relations (f = 3), suggesting the importance of strengthening communication and collaboration between schools and the wider community. As a solution, they emphasized the need for increased support from local administrations (f = 2), reflecting their belief that successful implementation of inclusive leadership requires stronger institutional and community-level support. Additionally, uninterested and inattentive students (f = 1) and communication problems with parents (f = 1) were identified as barriers to achieving desired educational outcomes. Overall, local authorities viewed inclusive leadership not only as a school-based practice but also as a collaborative process that depends on effective partnerships between schools, families, and local institutions.

**Figure 1:**
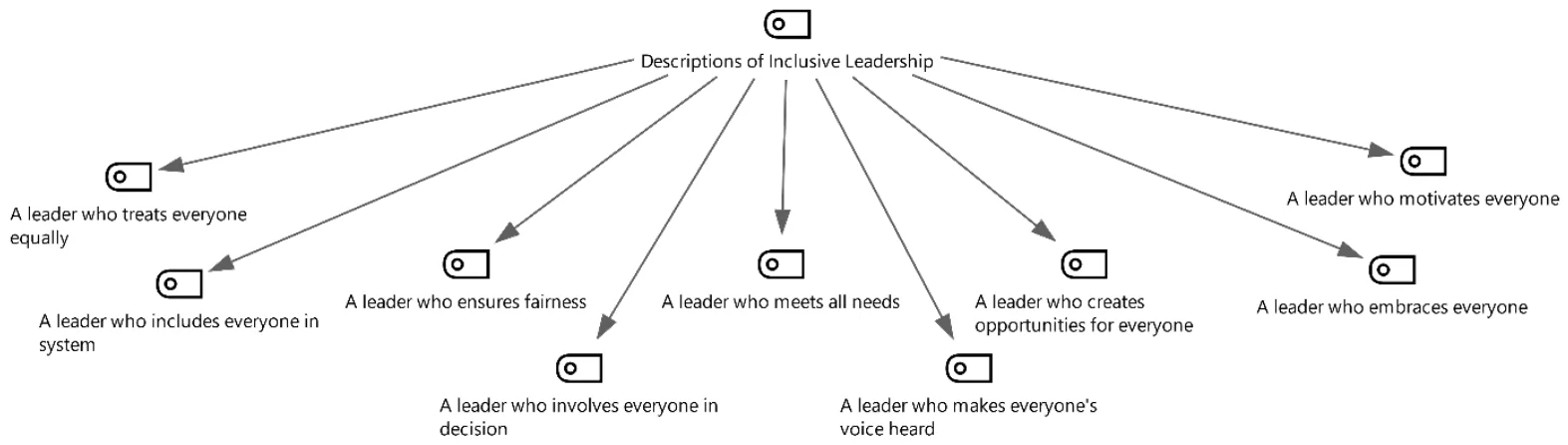
Hierarchical Code-Subcode Display of Descriptions of Inclusive Leadership.

According to Figure 1, the findings of the study indicate that the understanding of inclusive leadership in disadvantaged neighbourhoods is strongly perceived along the axes of “equality” and “participation.” The most frequently emphasised category by participants, “a leader who treats everyone equally,” reveals that the foundation of inclusive leadership lies in eliminating discrimination and ensuring a fair approach toward all stakeholders. In parallel, the high frequency of the expression “a leader who includes everyone in the system” suggests a strong awareness that inclusion should not be limited to individual attitudes but must also be reflected in institutional structures and processes. The similar frequency of the categories “a leader who ensures fairness” and “a leader who involves everyone in decision-making processes” indicates that perceptions of equality are associated not only with outcomes but also with fairness in processes.

On the other hand, categories such as “a leader who makes everyone’s voice heard” and “a leader who meets all needs” point more toward the communicative and supportive dimensions of inclusion; however, they are not perceived as central as equality and systemic inclusion. Lower-frequency expressions such as “a leader who embraces everyone” and “a leader who creates opportunities for everyone” suggest that the emotional and opportunity-related dimensions of inclusive leadership are relatively less emphasised. The least mentioned category, “a leader who motivates everyone,” indicates that motivation is not strongly recognized as a core component of inclusive leadership.

These findings are noteworthy in that they demonstrate how inclusive leadership is interpreted through different layers of meaning depending on stakeholders. School administrators tend to emphasize “a leader who treats everyone equally” and “a leader who includes everyone in the system,” suggesting that they approach inclusion from a more structural and managerial perspective. Within this framework, inclusion is associated with organizing rules, processes, and school functioning in a way that encompasses all individuals, that is, ensuring equality and fairness at the system level. This perspective may stem from administrators’ responsibilities, which require organisational order, policy-making, and the creation of structures that include all stakeholders.

In contrast, teachers emphasized equality, fairness, participation in decision-making, and responsiveness to stakeholders’ needs. Compared with administrators, they placed relatively greater emphasis on relational aspects of leadership such as listening to stakeholders and meeting diverse needs. As teachers are at the center of daily interactions within the classroom and school environment, they tend to prioritize leadership qualities that involve listening, responsiveness to needs, and making individuals feel valued. This suggests that teachers define inclusion not only as a systemic arrangement but also as a process grounded in interpersonal communication, empathy, and support.

Overall, these findings show that perceptions of inclusive leadership in disadvantaged neighbourhoods are largely concentrated on egalitarian approaches, the provision of justice, and the inclusion of all stakeholders within the system. This suggests that the primary expectation from leadership in such contexts is to reduce existing inequalities and eliminate the risk of exclusion. However, the relatively limited emphasis on more transformative elements such as motivation, individual development, and opportunity creation indicates that inclusive leadership tends to play a more “transformative potential” role in practice, while still holding the potential for a broader sphere of influence. Furthermore, the differences in perception between administrators and teachers highlight the multidimensional nature of inclusive leadership. While administrators focus more on structural and justice-based inclusion, teachers emphasize its human, emotional, and communicative aspects. These differing perspectives are, in fact, complementary. An effective inclusive leadership practice should therefore incorporate both system-level policies that ensure equality and participation, and a relational climate that listens to individuals, responds to their needs, and fosters a sense of belonging.

These findings also provide insight into how inclusive leadership may shape to academic achievement in disadvantaged school settings. Participants’ emphasis on equality, participation, fairness, and belonging suggests that inclusive leadership creates conditions that enable students and other stakeholders to feel valued and included in school processes. Such conditions may strengthen engagement with the school community and provide a foundation for improved learning outcomes. Furthermore, the coexistence of structural and relational dimensions of leadership indicates that academic success in disadvantaged contexts may depend not only on instructional practices but also on the creation of an inclusive and supportive school environment.

Direct statements of participants:

*A 9: “I define it as being able to adopt an equal and fair approach so that students, parents, teachers, and even other stakeholders can be peaceful and happy.”*

*T 16: “A leader who considers needs without discrimination, takes a fair approach to all relevant stakeholders, is solution-oriented, and believes that every idea is valuable.”*

*L 3: “I define it as employees or managers collectively making a joint decision on a matter, working together, and thus achieving success.”*

*A8: “For me, inclusive leadership is a democratic form of leadership built on the principles of empathy, equality, transparency, and collaborative learning, where everyone has a voice.”*

*T6: “Provide equal opportunities for all students, and to be more supportive of disadvantaged students.”*

**Figure 2:**
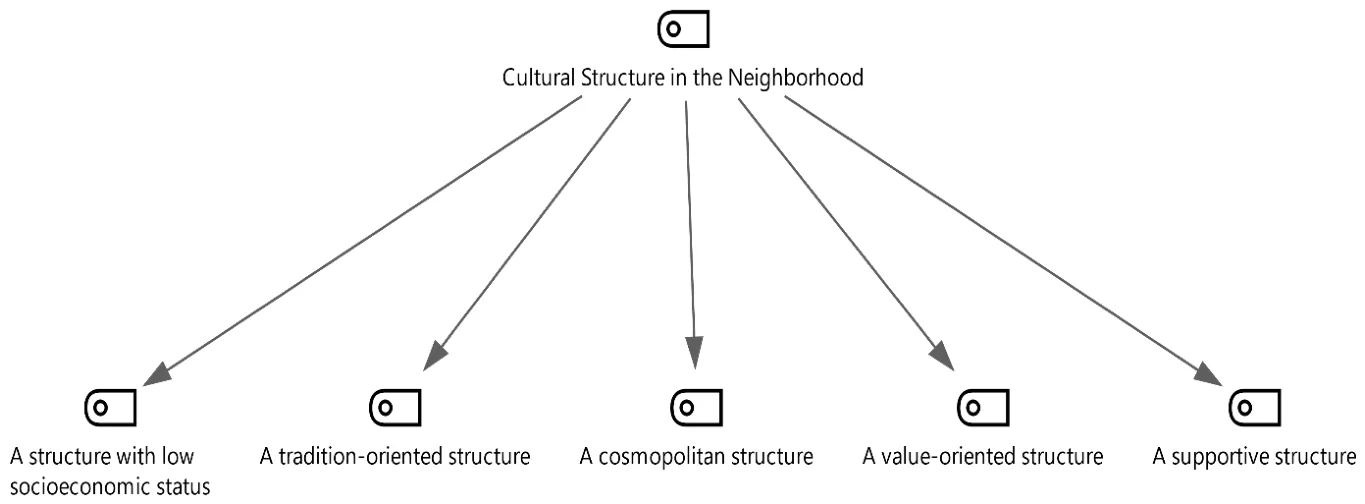
Hierarchical Code-Subcode Display of Cultural Structure in the Neighbourhood.

According to Figure 2, the findings indicate that the cultural structure of the neighbourhoods evaluated by the participants is largely shaped by socioeconomic conditions and traditional values. The category with the highest frequency, “a structure with low socioeconomic status,” reveals that these neighbourhoods are characterized by economic deprivation, limited resources, and disadvantaged living conditions. This situation also indirectly explains why inclusive leadership becomes more critical in such contexts; socioeconomic disadvantages increase inequality of opportunity in education and make the transformative potential of schools more significant.

The “tradition-oriented structure” category, which follows, shows that cultural norms, traditional values, and established social structures remain strong in these neighbourhoods. While such structures may support a sense of belonging and social solidarity, they may also create limitations in terms of openness to innovation and the adoption of inclusive practices. The relatively frequent mention of a “cosmopolitan structure” indicates that some neighbourhoods host diverse cultures, ethnic identities, or lifestyles, highlighting diversity as an important element. This diversity can be considered both an opportunity and a complexity that needs to be managed in the context of inclusive leadership practices.

On the other hand, the relatively low frequency of the categories “value-oriented structure” and especially “supportive structure” is noteworthy. The limited emphasis on a supportive structure suggests that participants did not frequently perceive social support mechanisms as a defining feature of the neighbourhood context.

Overall, these findings demonstrate that the cultural structure of disadvantaged neighbourhoods is multidimensional but predominantly shaped by low socioeconomic conditions and traditional values. At the same time, a certain degree of cultural diversity is also evident. This context provides a foundation that both complicates and necessitates the implementation of inclusive leadership practices. Leaders need to develop strategies to mitigate the effects of economic and social disadvantages while also managing traditional structures and cultural diversity in a balanced way to create a school climate that includes all stakeholders.

These findings further suggest that inclusive leadership may be particularly important in disadvantaged communities because school leaders are required to address challenges that extend beyond the school itself. Low socioeconomic conditions, cultural diversity, and traditional social structures create barriers that can negatively affect students’ educational opportunities and academic achievement. In this context, inclusive leadership appears to function as a mechanism for reducing the effects of exclusion and inequality by promoting participation, trust, and access to educational support.

Direct statements of participants:

*A 18: “Our school has a structure where religious and national values are strong, and where diverse socio-economic and cultural family structures coexist. Our students are raised with traditional values while striving to adapt to the modern world. This diversity is an important element that enriches the school culture.*

*L 2: “The academic performance of children in the neighbourhood is low, due to the family environment and the lack of parental oversight. The socioeconomic situation is poor.”*

*T 26: “There are people from very different cultures in the neighbourhood, and cultural degradation is a concern.”*

*A9: “It is a place with low socio-economic status and education levels, a large family culture, and a high concentration of children from working-class families.”*

*L2: “There aren’t enough resources for academic success. A school designed for 500 students has 2000. Morning and afternoon shifts are very crowded. In such an educational system, both the teachers and the school are insufficient for the number of students.”*

**Figure 3:**
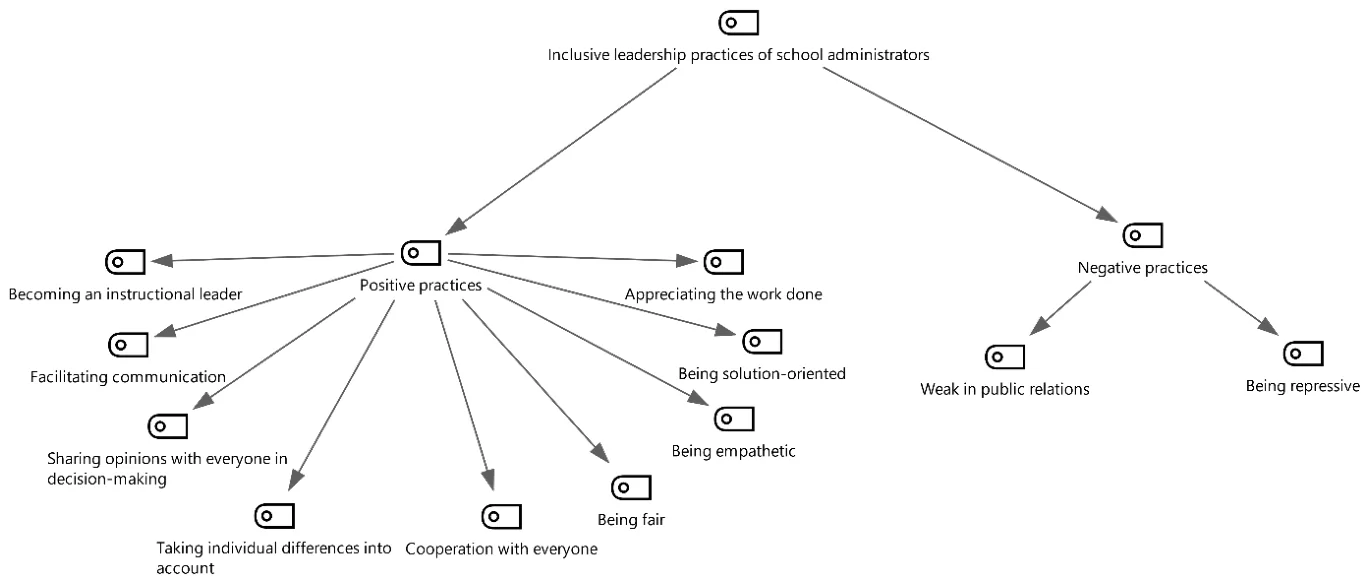
Hierarchical Code-Subcode Display of Inclusive Leadership Practices of School Administrators.

According to Figure 3, the findings indicate that school administrators’ inclusive leadership practices are largely shaped by positive approaches. In particular, the prominence of the instructional leadership role suggests that administrators are not limited to administrative tasks but are actively involved in teaching and learning processes and focused on supporting academic achievement. In addition, practices such as facilitating communication and valuing the sharing of opinions in decision-making processes demonstrate that the participatory and open communication aspects of inclusive leadership are strongly embraced. This indicates an effort to create a school climate in which different stakeholders can express themselves and contribute to the process.

Furthermore, practices such as considering individual differences, encouraging cooperation, being fair, and adopting a solution-oriented approach suggest that both the pedagogical and social dimensions of inclusive leadership have been substantially internalized by administrators. These practices play a critical role, especially in disadvantaged contexts, in fostering sensitivity to the diverse needs of students and teachers. However, the relatively limited emphasis on more emotional and motivational aspects, such as empathy and appreciating the work done, suggests that the relational dimension of inclusive leadership may require further development.

When negative practices are considered, the presence of issues such as repressive attitudes and weaknesses in public relations indicates that inclusive leadership is not fully implemented in all cases. The fact that these negative aspects are predominantly expressed by local community representatives suggests that the practices of school administrators are evaluated more critically by external stakeholders. This may indicate that, while inclusive practices within the school may be relatively strong, the same level of inclusiveness may not always be reflected in relationships with external stakeholders.

Overall, it can be concluded that school administrators demonstrate a largely positive orientation toward inclusive leadership. However, there remains room for improvement, particularly in areas such as empathy, appreciation, and effective communication with external stakeholders.

Importantly, the findings indicate that inclusive leadership practices contribute to academic achievement not only through direct instructional support but also through the establishment of participatory and supportive school processes. Practices such as encouraging collaboration, valuing diverse perspectives, and addressing individual needs appear to create conditions that facilitate student engagement and learning. This suggests that the potential role of inclusive leadership in achievement in disadvantaged contexts extends beyond traditional managerial responsibilities and involves building an inclusive learning environment for all stakeholders.

Direct statements of participants:

*L 4: “The time school administrators and teachers dedicate to their relationships with families and the public is less than one-thousandth of the time they spend playing card games in cafes. Most school administrators haven’t been able to extricate themselves from the grip of politics either. Their public relations are conducted within a political context.”*

*A 8: “I implement an inclusive leadership approach at school by ensuring the active participation of all stakeholders in the process. First and foremost, I establish open communication channels between teachers, students, and parents, and I make sure to listen to diverse perspectives when making decisions. I organize sharing meetings and in-service training sessions to support my teachers’ professional development; and I create environments where each student can express themselves through activities that consider their individual differences.”*

*T 7: “The school administration provides support education rooms for students with special learning needs, offers support in family meetings, and assists in providing time and space for additional studies.”*

*A14: “I hold regular open-door meetings with teachers. I value committee and teamwork in decision-making. I strive to develop support programs that take into account the individual differences of students. I establish mechanisms for transparent and regular communication with parents. I use a management style that includes teachers in the process by listening to their opinions.”*

*L2: “I think the school administration is weak in public relations. They need to get to the root causes of the problems and examine how to raise a quality individual.”*

**Figure 4:**
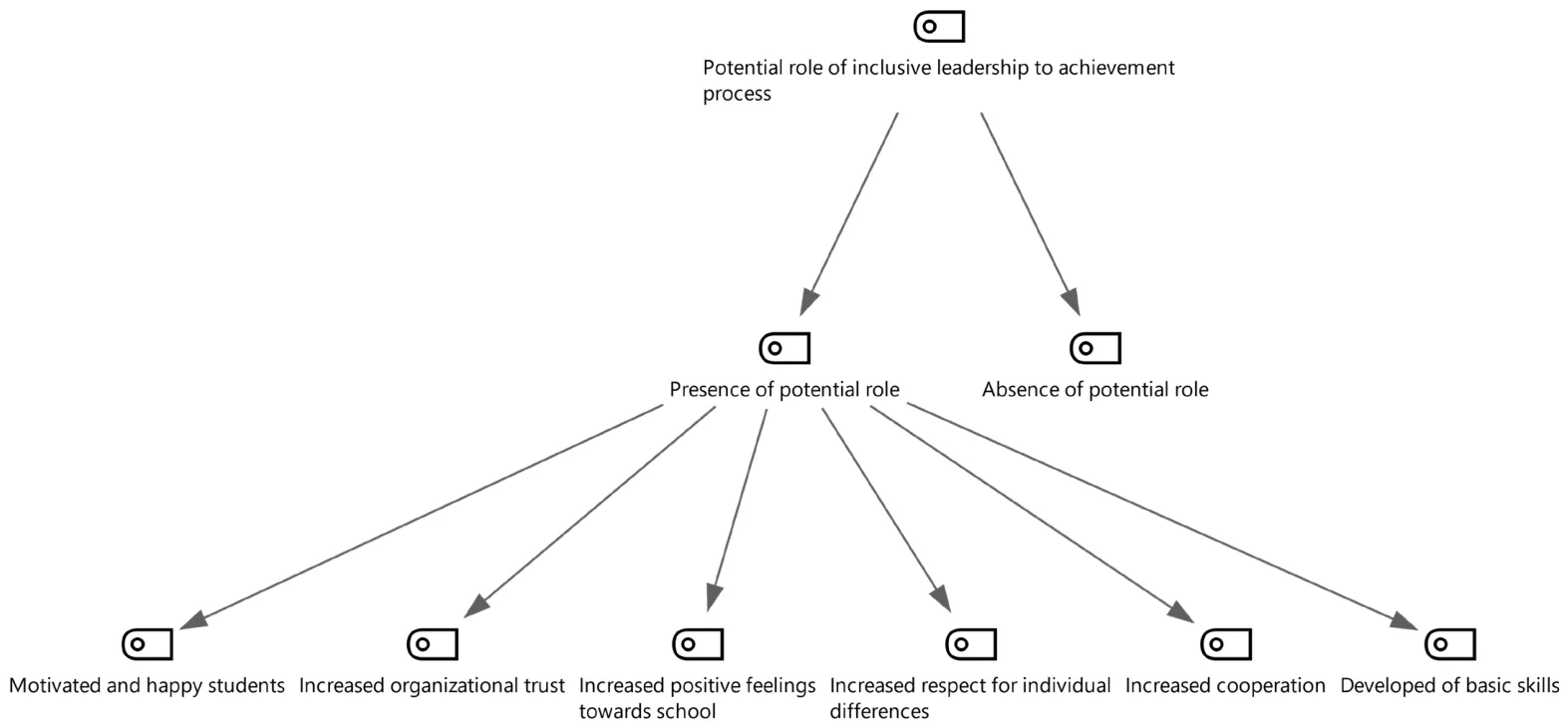
Hierarchical Code-Subcode Display of Potential Role of Inclusive Leadership to Achievement Process.

According to Figure 4, while most participants perceived inclusive leadership as having a positive role in the achievement process, a small number of participants (f = 3), all of whom were teachers, stated that they did not observe a meaningful contribution of inclusive leadership to academic achievement. The findings indicate that inclusive leadership makes generally positive and multidimensional contributions to the academic achievement process. In particular, the strong emphasis on increased student motivation and happiness suggests that inclusive leadership improves the emotional climate of the learning environment, which in turn indirectly supports academic performance. Similarly, the development of positive feelings toward the school and the increase in organisational trust point to the creation of a healthier, more supportive, and more collaborative environment among school stakeholders. Such a climate provides a critical foundation for the continuity and quality of learning processes.

In addition, findings such as increased respect for individual differences, strengthened cooperation, and the development of basic skills demonstrate that inclusive leadership is effective not only in emotional dimensions but also in social and academic domains. This indicates that inclusive leadership plays a significant role not only in students’ academic outcomes but also in their social adaptation and personal development.

On the other hand, it is noteworthy that some participants expressed the absence of a potential role, and these views were predominantly voiced by local community representatives. This suggests that while the effects of inclusive leadership are more visible and concretely experienced by internal school stakeholders, they may not be perceived to the same extent by external stakeholders. The tendency of local community representatives to focus more on macro-level outcomes and tangible results may lead them to perceive the indirect and long-term effects of inclusive leadership as limited. At the same time, this finding may also indicate that communication and collaboration between schools and external stakeholders are not sufficiently strong.

Overall, it can be concluded that inclusive leadership shapes the achievement process, particularly through motivation, trust, and the creation of a positive school climate. However, the fact that this potential role is not perceived equally by all stakeholders highlights the need to increase the visibility of inclusive leadership practices and to establish stronger relationships with external stakeholders.

Beyond identifying positive outcomes, the findings reveal a potential mechanism through which inclusive leadership contributes to academic achievement. Participants consistently linked inclusive leadership to increased school belonging, higher motivation, and stronger organisational trust. These factors appear to function as intermediate conditions that enhance students’ engagement with learning processes and ultimately support academic performance. This finding extends existing research by providing a qualitative explanation of how inclusive leadership may influence achievement in disadvantaged school contexts.

Direct statements of participants:

*L 4: “No… School administrators are not leaders. Perhaps they are managers. Leadership and management are different things. Leadership involves being able to reach out to everyone and embrace the masses. School administrators cannot go beyond implementing regulations…”*

*A 18: “I observe that our students’ sense of belonging to the school has increased, their self-confidence has improved, and their academic performance has improved. Furthermore, there has been a decrease in disciplinary problems and an increase in participation in social activities.”*

*T 15: “Thanks to the inclusive leadership implemented by our school administration, children with diverse abilities have begun to love school more and feel a sense of belonging. As a result, problems such as absenteeism have been eliminated.”*

*T19: “In inclusive leadership, students feel valued at school. When students with diverse abilities are supported by the school administration, there is solidarity rather than competition among students. This motivates them.”*

*T1: “I haven’t seen any reflective difference when comparing my 22 years of experience, past and present.”*

**Figure 5:**
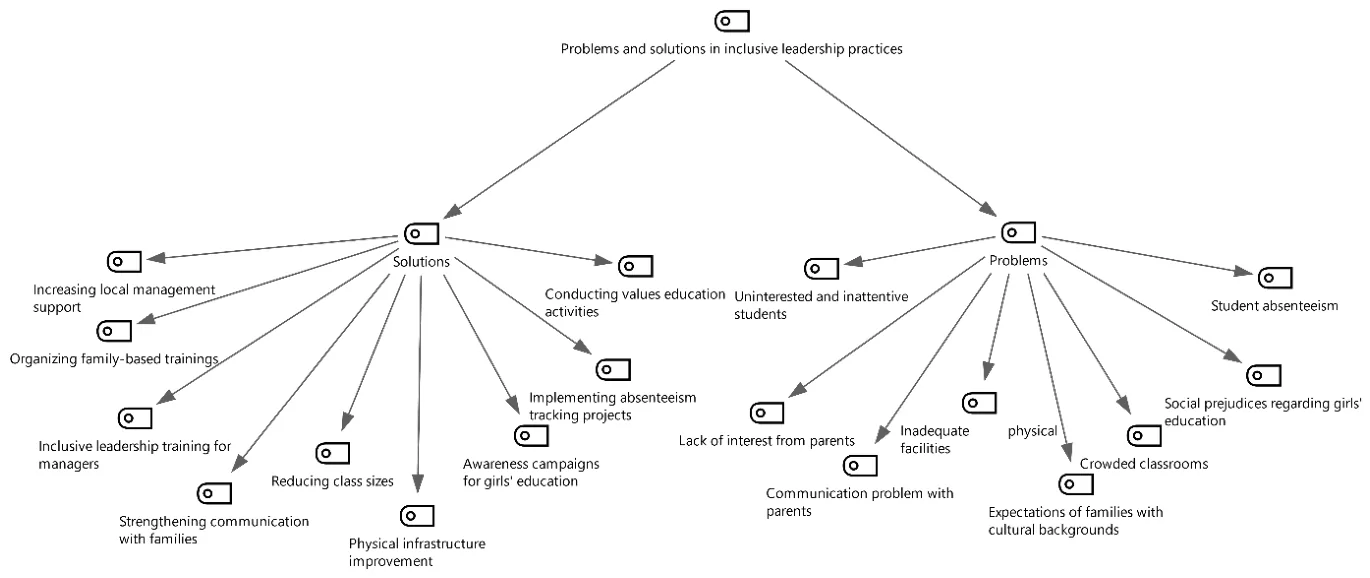
Hierarchical Code-Subcode Display of Problems and Solutions In Inclusive Leadership Practices.

According to Figure 5, the findings indicate that the problems encountered in inclusive leadership practices are largely concentrated around family, student, and structural factors, while the proposed solutions aim to address these multidimensional issues in a balanced way. When examining the problems, parental lack of interest and students’ lack of motivation or attention stand out prominently. This highlights the significant role of out-of-school learning environments and family support in academic achievement. Similarly, issues related to communication with parents and differences in expectations arising from diverse cultural backgrounds suggest that a strong, shared understanding and collaboration between schools and families has not been fully established. Structural challenges, such as inadequate physical facilities and classroom conditions, also emerge as important factors limiting the effectiveness of inclusive practices.

The proposed solutions, on the other hand, reflect a notably stakeholder approach. The strong emphasis on increasing support from local community representatives indicates that inclusive leadership should be addressed not only within schools but also within a broader ecosystem. Additionally, the expansion of family-based training programs and efforts to strengthen communication with families directly correspond to the identified problem areas, placing family involvement at the center of the solution. This underscores the critical role of families in both academic success and inclusivity.

Training programs on inclusive leadership for school administrators also emerge as a key solution area, suggesting that enhancing leadership capacity will play a crucial role in addressing existing challenges. Meanwhile, recommendations such as reducing class sizes and improving physical infrastructure are aimed at eliminating structural barriers. More specific interventions such as awareness campaigns for girls’ education, absenteeism tracking projects, and values education activities can be seen as complementary strategies targeting particular issues.

Overall, the findings demonstrate that the challenges in inclusive leadership practices are not limited to internal school dynamics but are closely linked to family, community, and structural conditions. Accordingly, the proposed solutions reflect a multidimensional approach, emphasizing the need to simultaneously strengthen leadership capacity, increase family involvement, and enhance support from local community representatives. This suggests that for inclusive leadership to be effective, schools alone are not sufficient; broader collaboration and coordination are essential.

The findings also demonstrate that the impact of inclusive leadership on academic achievement is shaped by contextual conditions. Family disengagement, economic hardship, inadequate educational resources, and limited community support emerged as factors that restrict the effectiveness of inclusive leadership practices. Therefore, in disadvantaged communities, academic achievement appears to depend not only on leadership practices within schools but also on the extent to which schools can mobilize support from families, local institutions, and the broader community. This highlights a context-specific dimension of inclusive leadership that has received limited attention in previous research.

Direct statements of participants:

*L3: “I believe it’s appropriate for teachers, especially classroom teachers, to inform each student’s family about their situation, behavior, and special skills. If there’s a problem, they should work together, like finding a diamond in a coal mine.”*

*T19: “We are having difficulty obtaining materials and equipment because the students’ families do not have good economic situations. This negatively affects our lessons.”*

*T15: “School administrators should prioritize teamwork to strengthen their inclusive leadership roles, create a collaborative environment within the school, emphasize social and cultural activities, support students’ socio-cultural development alongside their academic progress, and be open to creative and innovative ideas.”*

*A18: “I believe that we need to increase education-focused social projects, strengthen awareness campaigns regarding girls’ education, and ensure more active participation of local institutions in academic support programs.”*

*A3: “There are problems such as spoiled students, disrespectful parents, lack of manners, and overcrowded classrooms.”*

## 4. Discussion

This study’s findings indicate that the concept of inclusive leadership in schools located in disadvantaged neighbourhoods presents a multidimensional structure both in terms of how it is perceived and how it is practiced. First, the findings related to the definition of inclusive leadership show that participants predominantly interpret this concept through the lenses of inclusion in the system. This suggests that in disadvantaged contexts, the main expected function of leadership is to reduce existing inequalities and ensure the participation of all stakeholders in the educational process. However, the relatively limited emphasis on more transformative dimensions such as motivation, opportunity creation, and emotional support indicates that inclusive leadership tends to function more as a “transformative potential” mechanism in practice, while its potential is actually much broader. Inclusive leadership shares several characteristics with related approaches such as social justice leadership, culturally responsive leadership, distributed leadership, and transformational leadership. However, participants in this study particularly emphasized the active inclusion of diverse stakeholders in decision-making processes and the creation of a sense of belonging, suggesting that these aspects constitute central features of inclusive leadership in disadvantaged school contexts. In this respect, inclusive leadership appears to extend beyond improving organisational performance or distributing responsibilities by emphasizing voice, accessibility, and participation among groups that may otherwise remain marginalized. Inclusive leadership considers and cares about the views and expectations of its followers in decision-making processes (Nembhard & Edmondson, 2006). This finding is consistent with Ryan (2006b), who argues that inclusive and socially just leadership seeks to reduce inequalities and ensure equitable participation for all stakeholders. Similarly, Khalifa et al. (2016) emphasize that leadership practices in marginalized communities should focus on addressing systemic inequities and promoting inclusion. While previous studies have highlighted empowerment and advocacy as important dimensions of inclusive leadership, participants in the present study placed relatively greater emphasis on equality and fairness. This suggests that in disadvantaged neighbourhood contexts, inclusive leadership may be perceived primarily as a mechanism for addressing immediate inequalities rather than as a broader transformative strategy.

Differences in perception among stakeholders constitute another significant finding. School administrators tend to conceptualize inclusive leadership more in structural and managerial terms, emphasizing equality, systemic inclusion, and justice, which aligns with their responsibilities. In contrast, teachers highlight relational aspects such as communication, participation, and sensitivity to individual needs, reflecting their focus on the daily interpersonal dimensions of inclusive leadership. The local community representative subgroup, which consisted primarily of male religious officials, tended to adopt a more critical perspective, drawing attention particularly to negative practices. This suggests that inclusive leadership may not be equally visible or perceived as equally effective by the local community representatives included in this study. Overall, this highlights the multidimensional nature of inclusive leadership and the need to balance differing stakeholder expectations. The differences observed between internal stakeholders (administrators and teachers) and external stakeholders (community representatives) may reflect differing institutional positions and experiences. Administrators and teachers are directly involved in implementing school policies and practices and may therefore be more inclined to emphasize positive aspects of inclusive leadership. In contrast, the local community representatives participating in this study may evaluate leadership practices from a more external and critical perspective. These differences highlight the importance of considering stakeholder position when interpreting perceptions of inclusive leadership. These differences among stakeholder groups support the argument that leadership is socially constructed and interpreted differently depending on individuals’ roles and experiences within the organization. Similar findings have been reported by Leithwood (2021), who noted that educational stakeholders often evaluate leadership effectiveness through the lens of their own responsibilities and expectations. The present findings extend this perspective by suggesting that the local community representatives included in this study may evaluate inclusive leadership more critically than internal school stakeholders, highlighting the importance of considering external perspectives when assessing leadership practices. This finding also suggests that inclusive leadership should not be understood solely as a set of leadership behaviours but as a relational and socially negotiated process whose meaning varies across stakeholder groups.

Findings related to the cultural structure of the neighbourhoods demonstrate that context is a key determinant of inclusive leadership practices. In neighbourhoods characterized by low socioeconomic status, strong traditional values, and a certain degree of cultural diversity, schools are perceived not only as educational institutions but also as social balancing agents. In this context, inclusive leadership carries the responsibility of both mitigating socioeconomic disadvantages and managing cultural diversity in a harmonious way. However, the perceived weakness of social support mechanisms suggests that leadership needs to play a more active role in this area. This finding is consistent with the culturally responsive leadership perspective proposed by Khalifa et al. (2016), which emphasizes that leadership practices cannot be separated from the sociocultural realities of the communities in which schools operate. Similarly, Santamaría and Santamaría (2016) argue that effective leadership in marginalized communities requires sensitivity to local cultural and socioeconomic conditions. The current findings further suggest that in disadvantaged neighbourhoods, inclusive leadership is expected not only to support learning but also to compensate for limited social support mechanisms within the broader community.

Findings regarding inclusive leadership practices generally present a positive picture. School administrators’ adoption of instructional leadership roles, strengthening of communication, support for participatory decision-making processes, and consideration of individual differences contribute to the development of an inclusive school climate. However, there is a need for improvement in areas such as empathy, appreciation, and effective communication with external stakeholders. In particular, criticisms raised by local community representatives regarding repressive attitudes and weak public relations indicate that the external dimension of inclusive leadership needs to be strengthened. In Culha and Nazlı’s (2023) research, the inclusive leadership practices exhibited by school administrators to ensure the participation of refugee students in education were determined to be guidance and cooperation. These findings are consistent with Carmeli et al. (2010), who identified openness, accessibility, and availability as core dimensions of inclusive leadership. School administrators’ efforts to strengthen communication, encourage participation, and recognize individual differences reflect these dimensions. However, criticisms regarding weak communication with external stakeholders suggest that the accessibility dimension of inclusive leadership may not be fully realized in practice.

Regarding the potential role of inclusive leadership in academic achievement, the findings show that this leadership approach is particularly effective through motivation, school engagement, and organisational trust. Increased student happiness and motivation, more positive feelings toward school, and the development of a culture of collaboration support a positive learning environment essential for academic success. However, the perception among some stakeholders, particularly the local community representatives included in this study, that the potential role of inclusive leadership is limited may stem from the indirect and long-term nature of these effects. This also highlights the need to strengthen communication and collaboration between schools and external stakeholders. More specifically, the findings suggest a pathway through which inclusive leadership may influence achievement. Inclusive leadership was associated with stronger school belonging, higher motivation, increased trust, and reduced absenteeism. These factors appear to act as intermediate mechanisms that enhance students’ engagement with learning processes and create conditions that support academic performance. Although academic achievement was not measured directly in this study, the findings provide qualitative evidence regarding how leadership practices may shape educational outcomes in disadvantaged contexts. The findings partially support previous research suggesting that leadership influences student achievement indirectly rather than directly. Leithwood (2021) argues that school leadership affects academic outcomes through mediating factors such as school climate, teacher motivation, and organisational conditions. Similarly, the participants in this study associated inclusive leadership with increased motivation, trust, engagement, and positive school experiences rather than with immediate academic outcomes. Therefore, the findings qualify existing literature by suggesting that the relationship between inclusive leadership and academic achievement may operate primarily through psychosocial and organisational mechanisms in disadvantaged school settings. Rather than directly influencing achievement, inclusive leadership appears to operate by strengthening psychosocial conditions within schools, including belonging, trust, participation, and engagement. These mechanisms distinguish inclusive leadership from more performance-oriented leadership approaches by highlighting the importance of stakeholder inclusion as a precondition for educational improvement.

These findings can also be interpreted through the lens of psychological safety (Nembhard & Edmondson, 2006), which served as the theoretical foundation of this study. Participants frequently associated inclusive leadership with voice, participation, trust, belonging, and open communication. These characteristics are consistent with psychologically safe environments in which individuals feel respected, valued, and comfortable expressing their ideas and concerns. From this perspective, inclusive leadership may contribute to educational processes by fostering psychological safety among students, teachers, and other stakeholders, thereby strengthening engagement and participation in school life.

Finally, when problems and proposed solutions in inclusive leadership practices are considered together, it becomes clear that the process is not limited to internal school dynamics. Issues such as parental disengagement, communication problems, lack of student motivation, and physical inadequacies are key factors limiting the effectiveness of inclusive leadership. In response, proposed solutions reflect a multidimensional approach, including strengthening leadership capacity, increasing family involvement, enhancing local community representatives’ support, and improving structural conditions. In particular, the emphasis on family-based education and support from local community representatives underscores the need for strong cooperation among schools, families, and the broader environment for inclusive leadership to be effective. Culha and Nazlı (2023) showed in their research that the most common problems encountered by school administrations when including refugee students in educational activities were language difficulties, low academic achievement, absenteeism, and adaptation problems experienced by refugee students. According to Culha and Nazlı’s (2023) research, the employment of Syrian teachers, sports and cultural activities, organization of integration courses, and provision of interpreter support are suggested. These findings are consistent with previous studies emphasizing the importance of family and community engagement in promoting educational equity. Khalifa et al. (2016) argue that meaningful collaboration between schools and communities is a key component of inclusive and culturally responsive leadership. The present study reinforces this argument by demonstrating that parental disengagement and weak community support are perceived as major barriers to the successful implementation of inclusive leadership.

Overall, this study demonstrates that inclusive leadership is both necessary and effective in disadvantaged neighbourhoods; however, its impact can be enhanced only if structural, relational, and contextual dimensions are addressed simultaneously. Culha and Nazlı’s (2023) research showed that there is no exclusion of refugee students in Türkiye. They demonstrated that cultural and religious similarities, as well as the inclusive leadership practices exhibited by school administrators, are effective in preventing this situation. This study suggests that stakeholders perceive inclusive leadership as an important mechanism for supporting student engagement, motivation, and positive school experiences in disadvantaged neighbourhoods. Rather than demonstrating a direct causal effect on academic achievement, the findings indicate that inclusive leadership may contribute to the organisational and relational conditions that are commonly associated with improved educational outcomes.

A key contribution of this study is that it identifies the mechanisms through which inclusive leadership may support academic achievement in disadvantaged communities. Rather than influencing achievement directly, inclusive leadership appears to strengthen school belonging, motivation, trust, participation, and engagement, which in turn create conditions that support learning and academic success. Furthermore, the findings demonstrate that the effectiveness of inclusive leadership is shaped by contextual factors such as socioeconomic disadvantage, family involvement, and community support, highlighting the importance of examining leadership within its broader social environment. The study also contributes to the conceptual understanding of inclusive leadership by suggesting that, in disadvantaged school settings, inclusive leadership is not perceived merely as a managerial approach promoting equality and participation. Rather, it is understood as a contextually embedded process through which diverse stakeholders are recognized, included, and provided opportunities to influence educational processes. This perspective suggests that inclusive leadership overlaps with related approaches such as distributed, transformational, culturally responsive, and social justice leadership. However, participants particularly emphasized stakeholder voice, belonging, participation, and recognition of diversity, highlighting dimensions that appear especially salient in disadvantaged school settings.

## 5. Conclusions

This study provides an important framework for understanding both leadership perceptions and the academic achievement process in schools located in disadvantaged neighbourhoods. School administrators tend to adopt a more structural and system-oriented understanding of inclusion, whereas teachers interpret this process more through relational and emotional dimensions. The more critical perspective of local community representatives, on the other hand, suggests that inclusive leadership is not always equally visible or perceived in the same way by external stakeholders.

The findings suggest that inclusive leadership may be associated with factors such as student motivation, school engagement, and organisational trust, which participants perceived as contributing to academic achievement. However, these relationships are based on stakeholder perceptions and should not be interpreted as evidence of a causal effect. Furthermore, the perceived influence of inclusive leadership appears to be shaped by contextual conditions, including low socioeconomic status, traditional social structures, parental disengagement, and inadequate physical infrastructure.

In conclusion, inclusive leadership plays a critical role in reducing inequalities and ensuring the participation of all stakeholders in disadvantaged educational settings. However, for this leadership approach to be effective, it should not be limited to internal school practices alone. It is essential to enhance family involvement, strengthen collaboration with local community representatives, and improve structural conditions. The main findings of the study by Gómez-Hurtado et al. (2021) demonstrate the importance of leaders in fostering an inclusive and collaborative culture in classroom practices. These practices should focus on the knowledge and cultural capital of foreign students; organisational and pedagogical strategies should be developed based on the recognition and participation of the educational community; commitment to social justice should be emphasised; and a collaborative approach to diversity management together with a shared understanding of educational inclusion should be adopted.

Recommendations for policymakers: Training programs aimed at developing inclusive leadership skills can be systematically expanded and made widely available as in-service training. Additional resources and budget support for schools in disadvantaged areas can be increased to reduce structural inequalities. National and local policies can be developed to strengthen cooperation among schools, families and local community representatives. Long-term planning can be implemented to reduce class sizes and improve physical infrastructure. Awareness and monitoring programs for girls’ education and combating absenteeism can also be expanded. More specifically, structured school–community partnership mechanisms may be established in disadvantaged neighbourhoods. Rather than relying on informal interactions, schools could develop regular consultation forums that include local community representatives, parents, and school staff to identify context-specific barriers to student engagement and attendance.

Recommendations for practitioners: A participatory school culture can be established by involving all stakeholders in decision-making processes. Regular communication with parents can be maintained to ensure their active involvement in the educational process. Practices that enhance empathy, appreciation, and motivation within the school can be more widely implemented. Cooperation with local community representatives and external stakeholders can be strengthened to effectively utilise external support mechanisms. Given participants’ concerns regarding parental disengagement, schools may implement targeted outreach strategies for families who rarely participate in school activities. Home visits, small-group parent meetings, and community-based engagement initiatives may be particularly beneficial in disadvantaged neighbourhoods where traditional forms of parental involvement are often limited.

Since local community representatives expressed more critical views than internal stakeholders, schools may establish formal feedback mechanisms through which community representatives can regularly communicate concerns, expectations, and suggestions to school leadership. Such mechanisms may improve trust and increase the visibility of inclusive leadership practices beyond the school itself.

Because participants frequently associated inclusive leadership with school belonging and motivation, school leaders should prioritise practices that strengthen students’ sense of belonging. Mentoring programs, peer-support initiatives, and student participation councils may serve as practical tools for enhancing engagement in disadvantaged school settings.

Furthermore, the study suggests that inclusive leadership may be particularly significant in disadvantaged communities because it serves not only an educational function but also a compensatory social function. By reducing the effects of exclusion, limited opportunities, and weak social support, inclusive leadership helps create conditions that enable students to participate more fully in educational processes. This context-specific role extends current understandings of inclusive leadership beyond general school settings and highlights its importance in addressing educational inequalities. The findings suggest that the most pressing challenge in disadvantaged neighbourhoods is not merely the provision of resources but the creation of sustainable relational networks among schools, families, and local communities. Therefore, inclusive leadership should be understood not only as a school-based leadership approach but also as a community-engagement strategy capable of strengthening the social conditions that support educational participation.

### Limitations and Recommendations for Future Research

This study’s findings are important; however, they should be interpreted within certain limitations. First, the research was conducted only in a rural area in western Türkiye, which limits the generalizability of the results to different geographical regions, urban settings, or diverse sociocultural contexts. Similarly, the sample consisting of 5 local community representatives, 22 school administrators, and 26 teachers, although suitable for obtaining in-depth data in qualitative research, may not fully reflect all dimensions of inclusive leadership due to the absence of broader stakeholder groups such as students and parents. Although local community representatives were included to broaden stakeholder perspectives, their small number and relatively homogeneous background limit the diversity of views captured from the wider community. Furthermore, all community representatives were male and most held religious leadership roles. Therefore, the perspectives of women and other community actors may be underrepresented.

In addition, the use of only qualitative methods, particularly interviews, means that the findings are based on participants’ subjective perceptions and may include biases such as social desirability. Because the findings rely on self-reported perceptions, participants may have presented their experiences in socially desirable ways. In particular, administrators and teachers may have emphasised positive aspects of leadership practices due to their institutional roles and close involvement in school processes. Therefore, the findings should be interpreted as stakeholder perceptions rather than objective assessments of leadership effectiveness.

Relatedly, the study did not utilise objective indicators of student performance, such as grades, standardised test scores, graduation rates, or attendance records. Instead, conclusions regarding academic achievement are derived from the perceptions and experiences of adult stakeholders, including school administrators, teachers, and local community representatives. Therefore, the findings should be interpreted as reflecting participants’ perceptions of the potential role of inclusive leadership in academic achievement rather than providing direct evidence of a causal relationship between inclusive leadership practices and student outcomes.

Furthermore, the cross-sectional design of the study limits the ability to examine changes and the long-term effects of inclusive leadership over time. For future research, several suggestions can be made. First, similar studies should be conducted in different regions, particularly in urban areas and across varying socioeconomic contexts, to enhance the comparability of findings. Expanding the sample to include students, parents, and other educational stakeholders would contribute to a more comprehensive understanding of the multidimensional nature of inclusive leadership. In addition, mixed-method studies combining qualitative and quantitative data could help validate the findings more robustly. Future studies may also strengthen the evidence base by combining stakeholder perceptions with objective measures of academic performance. Longitudinal studies could provide deeper insights by examining the effects of inclusive leadership on academic achievement over time. Moreover, future research should focus more on less emphasised dimensions of inclusive leadership, such as motivation, emotional support, and opportunity creation, in order to develop more concrete recommendations in these areas. Finally, intervention-based studies aimed at strengthening school–family–community collaboration could make significant contributions to improving the practical impact of inclusive leadership.

## Figures and Tables

**Table 1:** Distribution of Demographic Characteristics of the Teacher Group.

Variables	Groups	f
Gender	Female	16
	Male	10
The period served in the neighbourhood	1–5 years	10
	6–10 years	9
	11–15 years	2
	16 years and above	5
Seniority	1–5 years	1
	6–10 years	2
	11–15 years	6
	16 years and above	17
Branch	Turkish Language Teaching	6
	Science Teaching	4
	Guidance and Psychological Counseling	4
	Technology Teaching	4
	Classroom Teaching	1
	Physical Education and Sports Teaching	1
	Social Studies Teaching	2
	Mathematics Teaching	3
	English Teaching	1

**Table 2:** Distribution of Demographic Characteristics of the Local Community Representatives Group.

Variables	Group	f
The period served in the neighbourhood	1–5 years	2
	6–10 years	1
	11–15 years	1
	16 years and above	1
Education Status	Undergraduate	3
	Postgraduate	2
Job	Religious official	4
	NGO president	1

**Table 3:** Distribution of Demographic Characteristics of the Administrator Group.

Variables	Groups	f
Gender	Female	9
	Male	13
The period served in the neighbourhood	1–5 years	14
	6–10 years	6
	11–15 years	2
Seniority	1–5 years	6
	6–10 years	5
	11–15 years	7
	16 years and above	4
School Type	Primary School	5
	Secondary School	7
	High School	10

**Table 4:** Teachers’ Perspectives on Inclusive Leadership.

Theme	Code/Sub-Code	f
Descriptions of Inclusive Leadership	A leader who treats everyone equally	6
	A leader who makes everyone’s voice heard	3
	A leader who ensures fairness	4
	A leader who creates opportunities for everyone	1
	A leader who meets all needs	4
	A leader who embraces everyone	1
	A leader who involves everyone in decision-making	4
	A leader who includes everyone in the system	3
Cultural Structure in the Neighborhood	A tradition-oriented structure	1
	A structure with low socioeconomic status	1
	A cosmopolitan structure	1
Positive Practices	Taking individual differences into account	4
	Sharing opinions with everyone in decision-making	3
	Cooperation with everyone	3
	Being solution-oriented	1
	Being fair	1
	Facilitating communication	5
	Becoming an instructional leader	10
Negative Practices	Being repressive	2
	Weak in public relations	1
Presence of Potential Role	Increased cooperation	1
	Increased respect for individual differences	3
	Development of basic skills	2
	Increased organizational trust	4
	Increased positive feelings towards school	3
	Motivated and happy students	12
Absence of Potential Role Solutions	Absence of potential role	3
	Inclusive leadership training for managers	8
	Reducing class sizes	3
	Strengthening communication with families	2
	Organizing family-based trainings	2
	Increasing local management support	1
Problems	Crowded classrooms	1
	Uninterested and inattentive students	5
	Expectations of families with cultural backgrounds	1
	Inadequate physical facilities	3
	Communication problems with parents	4
	Lack of interest from parents	12

**Table 5:** Administrators’ Perspectives on Inclusive Leadership.

Theme	Code/Sub-Code	f
Descriptions of Inclusive Leadership	A leader who treats everyone equally	7
	A leader who makes everyone’s voice heard	1
	A leader who ensures fairness	3
	A leader who motivates everyone	1
	A leader who creates opportunities for everyone	1
	A leader who meets all needs	1
	A leader who embraces everyone	1
	A leader who involves everyone in decision-making	2
	A leader who includes everyone in the system	9
Cultural Structure in the Neighborhood	A tradition-oriented structure	6
	A structure with low socioeconomic status	9
	A value-oriented structure	3
	A supportive structure	1
	A cosmopolitan structure	5
Positive Practices	Taking individual differences into account	4
	Sharing opinions with everyone in decision-making	6
	Cooperation with everyone	4
	Being solution-oriented	4
	Being empathetic	3
	Being fair	5
	Facilitating communication	5
	Appreciating the work done	1
	Becoming an instructional leader	1
Negative Practices	Being repressive	0
	Weak in public relations	0
Presence of Potential Role	Increased cooperation	3
	Increased respect for individual differences	3
	Development of basic skills	2
	Increased organizational trust	9
	Increased positive feelings towards school	7
	Motivated and happy students	7
Absence of Potential Role	Absence of potential role	0
Solutions	Inclusive leadership training for managers	0
	Reducing class sizes	0
	Awareness campaigns for girls’ education	1
	Implementing absenteeism tracking projects	1
	Strengthening communication with families	3
	Conducting values education activities	1
	Organizing family-based trainings	7
	Increasing local management support	13
	Physical infrastructure improvement	3
Problems	Crowded classrooms	0
	Social prejudices regarding girls’ education	1
	Student absenteeism	1
	Uninterested and inattentive students	3
	Expectations of families with cultural backgrounds	2
	Inadequate physical facilities	1
	Communication problems with parents	4
	Lack of interest from parents	7

**Table 6:** Local Community Representatives’ Perspectives on Inclusive Leadership.

Theme	Code/Sub-Code	f
Descriptions of Inclusive Leadership	A leader who treats everyone equally	2
	A leader who involves everyone in decision-making	1
	A leader who includes everyone in the system	1
Cultural Structure in the Neighborhood	A tradition-oriented structure	1
	A structure with low socioeconomic status	2
Negative Practices	Weak in public relations	3
Presence of Potential Role	–	0
Solutions	Increasing local management support	2
Problems	Uninterested and inattentive students	1
	Communication problems with parents	1

## Data Availability

The data presented in this study are available on request from the corresponding author.
